# The art of play: anchoring, creative apperception, and psychosocial wellbeing in a community arts centre

**DOI:** 10.3389/fpsyg.2026.1974117

**Published:** 2026-10-28

**Authors:** Michael Garbutt, Lydia Gitau, Jill Bennett

**Affiliations:** Big Anxiety Research Centre, School of Art & Design, Faculty of Arts, Design & Architecture, UNSW Sydney, Sydney, NSW, Australia

**Keywords:** anchoring, arts and health, creative apperception, cultural participation, experiential design, play, wellbeing

## Abstract

This article reports on a pilot workshop program, *Anchoring: Finding Your Centre*, delivered at Bankstown Arts Centre, Sydney, located in a culturally and linguistically diverse region experiencing elevated psychosocial stress associated with social isolation and dislocation. While arts institutions are increasingly framed as civic resources, many who could benefit do not identify as regular participants. This exploratory study forms part of a broader project investigating how playful, trauma-aware cultural practices can support capacities for managing stress, loneliness, and disconnection in everyday life. The program was built around the concept of anchoring—a felt sense of safety, connection and orientation that enables people to engage openly with their surroundings. Informed by Donald Winnicott’s theories of play and creative apperception, the program comprised three two-hour workshops integrating trauma-informed expressive practice with imaginative and embodied forms of engagement. Data consisted of facilitator observations, WHO-5 wellbeing surveys administered pre- and post-program, and follow-up interviews. Participants (*n* = 6) reported calm, relief from anxiety, creative renewal, and meaningful connection with others, spaces, and everyday objects. Four inter-related processes were identified (spatial orientation, imaginative projection, embodied grounding and relational play), each associated with a distinct form of anchoring: in place, in objects, in the body and in others. WHO-5 scores are reported descriptively; with only five participants completing both surveys, they cannot indicate change. Wellbeing appeared to emerge through relational modes of attention rather than any single activity, with several participants reporting continuing practices beyond the workshops. These findings suggest that cultural participation may support wellbeing not only through encounters with artworks, but through forms of attentional anchoring that participants can carry into everyday life.

## Introduction

Loneliness and social isolation are recognised as significant contributors to psychosocial distress, with implications for mental health, personal agency, and social participation across the life course (World Health Organization, 2023). Sustained disconnection from meaningful social and cultural engagement (as opposed to choosing solitude) has been associated with poorer mental-health outcomes and diminished wellbeing (Hawkley and Cacioppo, 2010; Holt-Lunstad et al., 2017). This is a psychosocial challenge that typically arises in the context of everyday pressures rather than as discrete clinical conditions, underscoring the need for responses that extend beyond formal mental-health services (Australian Institute of Health and Welfare, 2023).

As public, non-clinical spaces that can support reflection, meaning-making, and social connection (Fancourt and Finn, 2019), museums and arts centres are an important potential civic resource for addressing psychosocial wellbeing in everyday life. Importantly, evidence suggests that the contribution of cultural institutions to wellbeing depends not simply on the presence of art or cultural content, but on the forms of engagement they enable, shaping whether participation feels accessible, supportive, and meaningful (Sandell, 2007; Dawson, 2014; Chatterjee and Noble, 2013).

However, participation in museums and galleries remains unevenly distributed across the population. Regular cultural attendance is socially patterned, shaped by factors such as education, cultural familiarity, confidence, and prior experience, rather than by physical access alone (Sandell, 2007; Dawson, 2014). As a result, those who may benefit most from cultural spaces as resources for developing wellbeing are often least likely to see such institutions as welcoming to them. Addressing this gap requires both outreach and audience development, as well as programs that support the development of transferable capacities—ways of attending, relating, and making meaning that individuals can take up within cultural settings and apply beyond them in everyday life.

A range of museum- and gallery-based initiatives already demonstrate how cultural participation can support attentiveness, reflection, and presence. Programs centred on mindfulness, slow looking, contemplative engagement, and dialogic learning can deepen encounters with artworks and environments without requiring specialist knowledge or interpretive expertise (Tishman, 2017; Yenawine, 2013).

At the same time, such approaches often rely on reflective, verbal, or introspective modes of participation, which may not be equally accessible or inviting to all participants. This highlights the need for complementary forms of engagement that can accommodate different thresholds for participation, including those who may find sustained stillness, verbal reflection, or self-directed contemplation challenging. One response to the limits of reflective and contemplative approaches has been a growing interest in playful forms of engagement within museums and arts centres. Play is well established as central to learning, exploration, and meaning-making in children’s museum programming, where it is understood as a primary mode through which knowledge is constructed and curiosity sustained (Hein, 1998; Falk and Dierking, 2013). By contrast, the role of play in adult cultural participation has received less theoretical attention. In museum contexts, play has often been associated with informality, entertainment, or education for children, and has therefore been treated as peripheral to serious adult engagement—a tendency noted in critical discussions of play and cultural value (Sicart, 2014).

Increasingly, however, museum scholarship and practice have proposed play as a legitimate adult activity that encompasses both imaginative, mindful engagement and embodied, active participation (Burnham and Kai-Kee, 2011; Grenier, 2010). Kai-Kee et al. (2020) observe that play can be understood as operating across a spectrum that includes imaginative, dialogic, and reflective modes of engagement alongside bodily, performative, and participatory forms. Imaginative play supports meaning-making, projection, and reflective attention, while embodied play supports grounding, vitality, and non-verbal pathways into participation. Together, these modes offer adults flexible entry points into cultural experience, complementing contemplative approaches.

Winnicott's (1971) account of play helps explain why such engagement matters for wellbeing. For Winnicott, play takes place in an intermediate area of experience between inner and outer reality, the same area in which he locates cultural experience in adult life. It is “in playing and only in playing that the individual child or adult is able to be creative and to use the whole personality” (p. 54). The mode of perceiving that play opens up he calls creative apperception: the external world is experienced as at once found and, in part, created, so that objects and places are invested with personal meaning while remaining real and other. Winnicott contrasts this with compliance, a relation to the world as something to be fitted in with or adapted to, and argues that it is creative apperception “more than anything else that makes the individual feel that life is worth living” (p. 65).

Play also depends on conditions. Winnicott describes playing as “inherently exciting and precarious” (p. 52), and the intermediate space in which it occurs opens only where experience has led to trust (p. 103). Where that trust is lacking, attention narrows toward vigilance and the world is met with compliance. We use the term anchoring for the felt sense of safety, connection and orientation that makes play possible (Garbutt, 2018). Anchoring is thus a precondition for play, and play is the ground of creative apperception. For people living with stress, dislocation or loneliness, invitations to play are more likely to be taken up once a person feels anchored, whether in place, in the body, in objects, or in relation to others.

In this study, creative apperception serves as an analytic lens on process, and was not measured as an outcome. We looked for it in participants’ language and conduct through four indicators: objects or environments described as responsive or as addressing the participant; projection of self into an object while the object remains other; a shift from evaluative or performance-oriented attention toward spontaneity; and a felt continuity between self and surroundings.

### Aims and design

The workshop-based study we report here set out to examine how a range of playful forms of engagement in an arts-centre setting could help develop transferable skills that might be taken up in everyday life. Bankstown Arts Centre is located in South Western Sydney, a region characterised by cultural and linguistic diversity, ongoing migration, and levels of psychosocial stress above the New South Wales average (Australian Bureau of Statistics, 2021; South Western Sydney Primary Health Network, 2025). Community consultations in the region identify poor access to psychiatric care, and, for culturally and linguistically diverse residents, barriers to care that include language, cost, difficulty navigating services, and social isolation (South Western Sydney Primary Health Network, 2025). Known for its commitment to socially engaged, artist-led practice and for programming that emphasises inclusion, participation, and engagement with everyday experience rather than passive consumption of cultural content (City of Canterbury Bankstown, n.d.; Create NSW, 2019), the Centre does not hold a permanent collection. While temporary exhibitions were present during the workshop series, the program was designed to focus on objects and environments rather than artworks.

*Anchoring: Finding Your Centre* was designed by two members of the Big Anxiety Research Centre at UNSW Sydney. The program’s title draws on the concept of anchoring, understood as a felt sense of safety, connection and orientation that enables people to engage openly with their surroundings (Garbutt, 2018). It combined Gitau’s trauma-informed expressive practice, which emphasises safety, choice, pacing, and non-verbal participation, particularly in contexts shaped by migration and ongoing stress (Gitau et al., 2022, 2023), with Garbutt’s museum-based work which foregrounds playful, imaginative and embodied modes of attention (Garbutt, 2013; Garbutt et al., 2024). These practices were brought into relation through an attentional orientation informed by Winnicott’s concept of creative apperception, described above. In this framework, anchoring provides the felt safety within which play, and hence creative apperception, becomes possible as the basis for cultivating the attentional and relational capacities that participants might take with them into everyday life.

### Research approach and methodology

Bankstown Arts Centre staff provided access to an indoor studio space and the Centre’s garden area, supplied art supplies and refreshment and assisted with recruitment through community networks, social media, and the Centre’s mailing list. The call-out invited prospective participants to “explore mindful awareness of your sensations, thoughts, and feelings—to feel more settled, calm the waters, and make new connections”. Three two-hour workshops were offered free of charge. The study was approved by the UNSW Human Research Ethics Advisory Panel (HC9105) and all participants provided informed written consent.

Each workshop was co-facilitated by Gitau and Garbutt. In what follows, “the facilitators” refers to them and “we” to all three authors. Workshops integrated embodied and somatic awareness, expressive and collaborative drawing (solo and paired), guided movement, and open-air exploration in the Centre’s gardens. Activities were designed to cultivate attentiveness, curiosity, and relational engagement rather than artistic skill or products, and to support participation through both imaginative and embodied forms of playful engagement. The workshops included the following activities:

*Soles of the feet*: tracing and decorating outlines of the feet while recalling moments of stability or support.

*Prospect and refuge*: an open-air exercise inviting participants to locate positions in the garden offering both openness and shelter, followed by reflection on feelings of safety and exposure.

*The photographic gift*: working in pairs, participants enacted the roles of photographer and camera. The “camera,” blindfolded and guided by the “photographer,” was gently positioned so that, when the blindfold was lifted, the view before them became a gift—an image offered as a moment of surprise or curiosity. Roles were then swapped.

*Blind-contour drawing*: covering the drawing hand and surface with a cloth to reduce outcome anxiety and encourage spontaneity.

*Object dialogue*: engaging in imagined conversation with objects, asking and responding to questions as a way of exploring projection, attentiveness, and relational meaning.

*Follow the leader*: in groups of three, participants drew together on a three-by-one-metre sheet of paper. One participant led with a continuous line while the others pursued it closely, until a facilitator’s signal prompted a change of leader.

These activities were designed to mobilise specific psychosocial processes. Exercises such as Prospect and Refuge drew on spatial dynamics of safety and exposure to support a felt sense of orientation, agency, and belonging within the environment. Somatic and drawing-based practices (e.g., Soles of the Feet, blind-contour drawing) were intended to promote grounding, attentional flexibility, and non-evaluative engagement, reducing performance pressure while supporting curiosity and presence. Relational exercises (e.g., Object Dialogue, Photographic Gift) invited projection, reciprocity, and responsiveness, enabling participants to experience objects, spaces, and others as capable of response rather than as inert or instrumental.

The pilot therefore functioned as a testbed for examining how such processes—spatial orientation, imaginative projection, embodied grounding, and relational play—contribute to the development of inner relational capacities. Participant accounts offered initial indications of how these capacities supported feelings of being at home, of acting with agency within shared space, and of carrying relational modes of attention into everyday life.

### Data collection

At the beginning of the workshop series and again at the conclusion of the final workshop, participants completed the WHO-5 Wellbeing Index, a five-item self-report measure of subjective wellbeing over the preceding 2 weeks, scored from 0 to 100 (Topp et al., 2015). At the end of each activity, participants were invited to share reflections, which facilitators documented as part of the observation notes used in the analysis. Each workshop concluded with informal discussion and refreshments, providing space for conversation and social exchange. One week after the end of the program, a follow-up interview was conducted via phone, audio-recorded, transcribed, and analysed.

### Process analysis

The analysis followed the workshops as an aesthetic process, keeping participants’ responses in the context of the activities and settings that prompted them. Its materials were the facilitators’ observation notes, written during and immediately after each session; participants’ reflections at the end of each activity, recorded in those notes; and the five follow-up interview transcripts. The facilitators also debriefed together after each workshop.

Analysis moved between three levels. First, we took as a starting point what each activity was designed to invite (the form of attention, movement or relation it proposed), as described above. Second, the facilitators observed what occurred as participants took it up, attending to moments of shift in attention, bodily orientation, language and engagement with objects, spaces and one another. Third, we read participants’ later accounts in the interviews against these observations, noting where they confirmed, extended or departed from what the facilitators had seen.

These descriptions were read through Winnicott’s account of play and creative apperception. The facilitators each prepared an account of the processes they observed, then compared and discussed them. The four processes reported below emerged from this comparison. Each process also corresponded to a distinct form of anchoring (in place, in objects, in the body, and in others). Table 1 links each process to the activities that invited it, what participants reported, and the corresponding indications of creative apperception. Participants are identified by gender and number (F1–F4, M1–M2).

**Table 1:** Processes identified, the activities that invited them, the forms of anchoring they supported, and indications of creative apperception.

Process	Activities	Anchoring	Participant reports	Creative apperception
Spatial orientation	Prospect and refuge; open-air exploration	Anchoring in place	Felt safety and belonging in the centre’s spaces; attention turning outward: “I realised the world carried me” (F4)	The world experienced as holding or supportive
Imaginative projection	Object dialogue; The photographic gift	Anchoring in objects	A candle holder that “gave me a sense of security” (M1); reading oneself through an object (F4); “using that as an anchor to the present” (F3)	Projection into objects; objects experienced as responsive
Embodied grounding	Soles of the feet; blind-contour drawing; guided movement	Anchoring in the body	Calm and relief from pressure (F3); freedom in movement (M1); loosened boundaries of self (M2)	Self and environment experienced as continuous
Relational play	Follow the leader; paired and group sharing	Anchoring in others	“Creativity and connecting with others” as personal anchors (F1); sharing stories (M1, M2, F4); WhatsApp group set up by participants	Others experienced as responsive

### Researcher positioning

The program was designed and co-facilitated by two of the authors. Garbutt is a design researcher whose museum-based practice centres on playful, contemplative and embodied engagement; Gitau is a trauma psychologist whose expressive-arts practice has focused on communities affected by migration and ongoing stress. Bennett, the director of the Big Anxiety Research Centre, critiqued successive drafts of the analysis and interpretation.

We regard the facilitators’ involvement as central to the method. In aesthetic and practice-led research, the facilitator is well placed to notice when the quality of a participant’s engagement shifts and to investigate its meaning. As designers of the program, the facilitators had an investment in its value, and participants may have been inclined to describe their experience favourably to people they had come to know. The facilitators’ own expectations may also have shaped what they noticed.

We held our interpretations to account in several ways. Each facilitator prepared an independent account before the two were compared. Follow-up interviews conducted by Garbutt and Gitau took place a week after the program, by phone and outside the workshop setting. In reading the interviews against our observations, we noted where participants’ accounts extended or departed from what the facilitators had seen, and we looked for descriptions of difficulty or ambivalence, but found none.

### Wellbeing measure

WHO-5 scores are reported as individual scores only. With five complete responses, they cannot indicate change at group level and carry little interpretive weight; no means or inferential tests are presented.

### Safeguards

The workshops followed trauma-informed principles of safety, choice, collaboration and pacing (Substance Abuse and Mental Health Services Administration, 2014). They were framed as cultural participation rather than therapy: participants were not asked to disclose or revisit distressing experiences, and prompts invited present-moment attention rather than personal history. At the start of each workshop, facilitators explained that any activity could be modified, observed or skipped without explanation, and that participants could leave the activity space at any time.

The Photographic Gift involved being blindfolded and physically guided, so it received additional safeguards. Facilitators described the activity in full beforehand and sought verbal consent from each participant. Participants could keep their eyes closed instead of wearing a blindfold, take only the guiding role, or observe.

A distress protocol was approved as part of the ethics application. If a participant showed or reported distress, Gitau would check in privately, offer a quiet space, and provide information on support services, including Lifeline (13 11 14). No distress events were observed or reported during the program.

## Results

Twelve people submitted expressions of interest, of whom six attended the first workshop; reasons for non-attendance among the other six were not collected. Eligibility required participants to be 18 or over and able to take part in English, and the expression-of-interest form invited applicants to explain why they wished to attend; no other screening was applied. Five participants attended all three workshops in person. The sixth (Participant 4) joined the final workshop online. The group comprised four women and two men; five were from non-English-speaking backgrounds (Chinese, Arabic and Malay), and four were university students. In their expressions of interest, several participants described experiences of anxiety, depression and burnout. Because participants self-selected in response to a call-out framed around calm, settledness and connection, the group is likely to have included people already open to reflective and creative activity. The accounts that follow describe this group’s experience and are not assumed to be representative of the wider community.

All six participants completed the WHO-5 at the first workshop. Five completed it again at the end of the final workshop, with the exception of Participant 4, who joined the session online. Five participants took part in the follow-up interview: Participant 2 did not. All participants contributed reflections during the workshops they attended.

Of the five participants who completed the second survey, three recorded higher scores, one fell, and one was unchanged (Table 2). With so few responses and no comparison group, these scores cannot indicate change and are reported for transparency only.

**Table 2:** WHO-5 wellbeing scores (0–100) at the start and end of the workshop series.

Participant	Score at start of workshops	Score during final workshop
1	20	48
2	48	36
3	24	24
4	68	–
5	56	92
6	44	52

– = not completed.

The scores are more informative as context. At the start, four of six participants scored below 50, the WHO-5 threshold for poor wellbeing, and two scored at or below 28, the level at which screening for depression is recommended (Topp et al., 2015). This is consistent with the stress, anxiety and burnout several participants described in their expressions of interest, and suggests the program reached people for whom wellbeing was a concern. Participants’ own accounts, reported below, are therefore the primary source for understanding their experience of the program.

Post-workshop interviews highlighted experiences of reduced anxiety, increased calm, and meaningful connection with others. As M1 reflected, the sessions offered “a nice, calm and open atmosphere… being able to share each other’s stories and learn from each other.” F3 described the workshops as her “meditation hour”, which helped her manage anxiety and depression: “It really did help me… it takes off the pressure.”

Participants also described shifts in how they experienced their own bodies, attention, and relation to surrounding space. M1 noted the freedom of drawing “and just letting the movement [take over]… that was freeing”, while M2 described blind-contour drawing as “a magical thing… it’s upgraded the boundaries of myself to the environment”. For F1, the workshops clarified that her personal anchors were “creativity and connecting with others… if I don’t have those two things in my life, I feel very down and sad”. Dialogue and story-sharing were valued, with M2 describing the experience as “receiving and responding to everyone”, and F4 observing, “I could find that my own questions also happened in others”. At the end of the workshop series, participants independently chose to establish a WhatsApp group in order to continue conversations and share reflections beyond the facilitated sessions.

Several participants reported that the practices had given them a renewed sense of agency. F3 explained that she had acquired a sense of “control… not the control of having to manage everything, but control in knowing that I am here, and whatever happens is okay.”

Objects and environments emerged as significant anchors for participants, supporting new forms of attentiveness and a sense of security. F4 explained that object dialogue altered her perception: “Reading the object, I read more about myself because I could project myself into the environment”. M1 recalled connecting with a candle holder—randomly selected from among various objects stored in the studio—describing how it “gave me a sense of security… being able to take things like that around the space and have it as a reassurance.”

Participants frequently described the program as a means of shifting from inward rumination toward outward engagement with the world. F4 explained that after a difficult personal rupture she “stopped looking inside myself and started to look outside… I realised the world carried me”. F3 described letting go of uncertainty through dialogue with objects and environments, noting that “letting go was my anchoring.”

Several participants reported continuing these practices independently after the workshop series ended. M1 described a bushland walk in which he was “focusing in, capturing details and seeing if I could feel either a connection or hear a whisper of what the boulders were saying”. F3 described integrating drawing and breathing into her daily routine: “I put these techniques into my practice… it evaporates into my body” and that “having conversation with objects… using that as an anchor to the present” had become part of her everyday life.

## Discussion

Participants’ accounts suggest that the workshops invited forms of relational engagement. Wellbeing appeared to emerge through capacities participants developed for attending, responding, and relating—to their own experience, to one another, and to the surrounding world. Psychosocial benefit appears to lie in these relational modes of engagement rather than the nature of the objects themselves.

A substantial body of arts and health research shows that encounters with artworks can support emotional regulation, reflection, meaning-making, and social connection (Fancourt and Finn, 2019; Chatterjee and Noble, 2013), but the Bankstown pilot suggests that comparable experiences may also arise through engagement with spaces, objects, and environments. This raises the possibility that arts centres without permanent collections may support wellbeing and that art museums might attend more explicitly to how space, objects, and embodied engagement mediate encounters with art or even constitute objects of attention in their own right.

Grounded in a felt sense of security, playful practices invited creative apperception that several participants reported carrying beyond the workshops, as reflected in reports of continued practice after the program concluded. Positioning play as central to this process aligns with research linking adult play to curiosity, creativity, flexibility, and social connection (Brown and Vaughan, 2009; Mainemelis and Ronson, 2006; Yogman et al., 2018), while museum scholarship notes that play-based initiatives remain largely directed toward children and families (Paris, 2017; Holdgaard and Olesen, 2023). In the Bankstown program, play operated as a relational stance rather than entertainment or competition: a shared, improvisational mode of engagement that supported curiosity, care, and responsiveness. As Sicart (2014) argues, play can be understood as an ethical stance toward others and the world; for several participants, this stance appeared to alter perception itself, enabling environments and objects to be experienced as responsive and alive.

The spatial and material dimensions of the workshops were integral to these shifts. In line with Dewey’s (1934) critique of the “museum conception of art” and scholarship on everyday aesthetics (Shusterman, 2012; Saito, 2017), aesthetic experience here emerged through attention to ordinary environments—paths, thresholds, trees, walls, doorways, and gardens—through which, for these participants, the arts centre became a site of felt safety. While the workshops share affinities with expressive-arts approaches in their use of embodied and imaginative practices (Malchiodi, 2020), they were framed as cultural participation rather than clinical intervention, oriented toward perception, attention, and connection rather than therapeutic change (Winnicott, 1971; Bennett et al., 2023; Bennett et al., 2024). Participants’ accounts of recognition and renewal resonate with Bollas’s (1992), pp. 78–79 notion of a “topographic return”, in which inner and outer landscapes are revisited and transformed through experience.

Taken together, and acknowledging the significant limitations of a small pilot study, the findings point to a possible role for arts centres and museums as civic spaces for cultivating transferable capacities of attention, play, and relational engagement, rather than as sites for the delivery of wellbeing outcomes alone. For museums with collections, where artworks are privileged anchors for reflection and connection, the findings suggest that playful, embodied modes of engagement attentive to space and materiality may lower barriers for audiences who feel excluded by specialist or language-based interpretive conventions (Kai-Kee et al., 2020; Hooper-Greenhill, 2000; Packer and Ballantyne, 2016). This orientation also resonates with the function of the ancient Greek *museion*, which was not a museum in the modern sense of a site for exhibitions, but a space dedicated to the Muses. To be inspired (*in-spirare*) was to breathe in an animating force, understood as a relationship between human attention and the sources of creativity.

### Scope of study

This study reports one program at one site, with six self-selected participants, five from non-English-speaking backgrounds and four of them university students. It describes how these participants engaged with the program over three sessions and in the week that followed. It cannot show whether similar processes would arise for other groups, in other settings or over longer periods, and reports of continued practice come from a single follow-up interview one week after the program. A larger study across several sites, with longer follow-up and participants recruited through trusted community settings, would allow these processes to be examined more widely.

## Conclusion

Recent preprint research indicates that encounters with original artworks in gallery settings can reduce stress and support wellbeing (Worrell et al., 2025), underscoring the established value of cultural participation and engagement with art as a psychosocial resource. Our Bankstown study complements this work by suggesting that the wellbeing potential of cultural participation lies not only in particular objects of focus, but in the internal capacities it cultivates. In the pilot, these capacities took the form of relational, playful, and trauma-aware ways of attending that several participants reported carrying into everyday life. At a time of heightened psychosocial stress and widespread experiences of being “alone together” (Turkle, 2011) arts centres and museums may have an important civic role to play. By inviting creative apperception, arts centres can contribute to the conditions through which meaning, connection, and belonging can be renewed in everyday life.

From this perspective, arts centres and museums can support wellbeing not only by facilitating engagement with artworks, but by attending to how people are invited to engage with spaces, objects, and one another. This includes creating accessible points of entry for those who may not identify as regular cultural participants, while enabling existing audiences to deepen their engagement and, where desired, move toward new forms of connection. Reaching adults who do not currently participate in cultural institutions will likely require invitations mediated through trusted community settings, such as neighbourhood organisations and social clubs.

## Data Availability

The datasets presented in this article are not readily available because they contain confidential participant interview data. Requests to access the datasets should be directed to the corresponding author.
